# Perceived exertion for guiding and monitoring exercise intensity in unsupervised, home-based cancer rehabilitation: an analysis of 3533 exercise sessions

**DOI:** 10.1186/s12885-026-15588-0

**Published:** 2026-01-20

**Authors:** Johannes Voß, Christian Leps, Ines Gockel, Uwe Tegtbur, Martin Busse, Roberto Falz

**Affiliations:** 1 Institute of Sports Medicine and Prevention, Leipzig University, Leipzig, Germany; 2 Clarunis – University Digestive Health Care Center, St. Clara Hospital and University Hospital Basel, Basel, Switzerland; 3 Department of Rehabilitation and Sports Medicine, Hannover Medical School, Hannover, Germany; 4 Department of Engineering and Industrial Design, Chair of Human-Machine Interaction, University of Applied Sciences Magdeburg-Stendal, Magdeburg, Germany

**Keywords:** Cancer survivors, Colorectal cancer, Prostate cancer, Breast cancer, Unsupervised home-based exercise, Telerehabilitation, Perceived exertion, Exercise oncology

## Abstract

**Background:**

Exercise therapy is an essential part of cancer rehabilitation. Home-based, unsupervised exercise programs supported by digital tools offer a promising way to reduce access barriers. However, guiding and monitoring exercise intensity without supervision remains difficult. Rating of perceived exertion (RPE) is often used for this purpose, but its validity in unsupervised, home-based settings has not been systematically examined. In this analysis, we evaluated whether RPE can guide and reflect objective exercise intensity, and explored factors influencing their alignment.

**Methods:**

We conducted a secondary analysis using data from the intervention group of a multicenter randomized controlled trial. Seventy-six cancer survivors (breast, colorectal, or prostate cancer) followed a six-month, unsupervised, home-based, video-guided exercise program (2 × 30 min/week of individually tailored strength-endurance with different body-weight exercises). Heart rate (HR) during training sessions was recorded via chest strap. After each session, participants reported RPE for overall intensity using the Borg category-ratio 10 scale. We used mixed-effects models to examine the relationship between RPE and session-mean relative exercise intensity (%HRmax), and to identify factors including age, sex, beta-blocker use, and training week (the sequential week of the intervention), influencing discrepancies between perceived and HR-based intensity. Cross-tabulations assessed how accurately RPE identified sessions within guideline-recommended intensity ranges.

**Results:**

In total, 3533 exercise sessions were analyzed. RPE was positively associated with %HRmax (B = 1.64, *p* < 0.001, marginal R² = 0.066, intraclass correlation coefficient = 0.60). RPE identified sessions within guideline recommendations with a sensitivity of 87.4% and a specificity of 22.3%. Discrepancies between perceived and HR-based intensity were systematically associated with age (B = 0.037, *p* < 0.001), sex (B = 0.64, *p* < 0.001), beta-blocker use (B = − 0.39, *p* < 0.001), and training week (B = 0.032, *p* < 0.001).

**Conclusions:**

RPE may help guide exercise intensity in unsupervised, home-based training for cancer survivors, but its limited alignment with physiological load may make it insufficient for precise monitoring. Objective feedback, such as HR monitoring, is needed to ensure adequate intensity in unsupervised settings. Future studies should explore factors influencing perceived exertion and test RPE across different exercise modalities.

**Trial registration:**

DRKS-ID: DRKS00020499; Registered 17 March 2020; https://drks.de/search/en/trial/DRKS00020499.

**Supplementary Information:**

The online version contains supplementary material available at 10.1186/s12885-026-15588-0.

## Introduction

Exercise has emerged as a core component of the cancer care continuum [1]. In cancer survivors, exercise can help to reduce treatment-related side effects, such as postoperative complications, fatigue, and muscle loss. It also improves quality of life and may lower the risk of recurrence and mortality [1–3]. Effective exercise prescription for cancer-related health outcomes includes moderate-intensity aerobic training three times per week for 30 min, alone or combined with resistance training twice per week. While resistance training contributes to functional recovery, its isolated effects on cancer-related outcomes are less consistent [4]. However, contextual and organizational barriers, such as travel distance, time constraints, financial efforts, and treatment-related discomfort, hinder patients from participating in regular supervised exercise [5–7].

Home-based exercise programs supported by digital tools have emerged as a promising alternative in cancer rehabilitation [8–10]. While evidence for their effectiveness is encouraging, findings remain heterogeneous across outcomes and cancer types [11, 12], though most studies report improvements in quality of life and functional capacity [8, 10, 11]. Such programs have been shown to be feasible, safe, and well accepted [13–18]. Their potential is particularly pronounced in unsupervised settings, where patients can train flexibly and independently without the need for direct supervision. However, when patients exercise independently, maintaining guideline-recommended intensity, essential for both efficacy and safety, becomes a major challenge. Moreover, adherence is critical for effectiveness. A meta-analysis found that when adherence exceeds 80%, differences in outcomes between home-based and supervised interventions become negligible [19].

The heart rate reserve and the percentage of maximum heart rate (%HRmax) are two commonly used methods for prescribing relative exercise intensity to improve cardiorespiratory fitness, with %HRmax used in the present study [20]. Current exercise guidelines recommend that cancer survivors should aim for an intensity of at least 60% HRmax [4, 21]. To meet this recommendation, home-based programs often rely on real-time heart rate (HR) monitoring via chest straps or wearables [11]. However, this approach requires equipment and repeated on-site testing, which limits its scalability and broad implementation.

Subjective rating of perceived exertion (RPE) offers a low-cost and easy-to-use method to prescribe and monitor exercise intensity [22, 23]. The Borg CR10 scale is a widely established tool to reflect RPE in exercise settings [24–27]. Several studies in controlled exercise settings show strong alignment between RPE and physiological intensity markers, supporting its general use in exercise prescription and monitoring [26–32]. Although RPE is already used in exercise oncology studies [11, 15], most evidence supporting its validity comes from cardiac rehabilitation, where patients similarly show complex health profiles and variable responses to exertion [33–35]. Its validity in unsupervised, home-based exercise programs for cancer survivors has yet to be systematically examined.

Therefore, we aimed to (1) test whether RPE can effectively guide and reflect HR-based exercise intensity during unsupervised, home-based training in cancer survivors after curative treatment, and (2) identify factors that explain discrepancies between perceived exertion and HR-based intensity.

## Methods

### Study design and participants

This secondary analysis draws on data from the intervention group of the Colorectal, Breast, and Prostate Cancer – Telemonitoring and Self-management (CRBP-TS) trial, a multicenter randomized controlled trial that evaluated a six-month home-based digital exercise and lifestyle program for cancer survivors following curative treatment. The full study protocol and first results have been published previously [18, 36, 37].

Participants were recruited between 2020 and 2021 from three German University Hospitals. Eligible individuals had undergone R0 resection for colorectal cancer (ICD-10: C18–C20), breast cancer (ICD-10: C50), or prostate cancer (ICD-10: C61), and were between four weeks and six months post-surgery at enrollment. Additional inclusion criteria were age 18–75 years, ECOG performance status ≤ 1, and sufficient cognitive and technological ability to participate in a digitally delivered intervention. Patients with severe comorbidities, active secondary malignancies, or lacking internet access were excluded.

For the current analysis, we included all patients from the intervention group (*n* = 76), who had participated in the home-based exercise training with HR monitoring, resulting in a total of 3771 recorded training sessions over the six-month intervention period. All participants provided written informed consent. The trial was approved by the Ethics Committee of the Medical Faculty of the University of Leipzig, Germany (reference number 056/20-ek) and registered in the German Clinical Trials Register (DRKS00020499).

### Exercise intervention

Participants in the intervention group received access to a structured, home-based exercise program delivered via the CRBP-TS app. The intervention followed a six-month strength-endurance protocol with weekly updated, asynchronous video sessions. Participants were encouraged to complete at least two sessions per week over the 24-week intervention period. Each session lasted approximately 30 min and included a warm-up, four rounds of circuit-style bodyweight exercises (e.g., squats, stepping, pushing, core work), and a cool-down. Exercises were performed in a time-based format (40 s of work, 20 s of rest) rather than a fixed number of repetitions, allowing participants to self-pace their movement speed and intensity according to perceived exertion. Between rounds, participants rested for 60 s and received recovery instructions (e.g., to walk in place, to drink). No formal stop criteria were defined based on HR or RPE. However, participants were advised through the video instructions to reduce intensity if their perceived exertion exceeded 8 on the Borg CR10 scale. Participants were instructed to interrupt the session if they experienced pain, dizziness, or any unusual discomfort.

Each exercise video featured both an instructor and a patient performing the exercises simultaneously. The instructor provided real-time verbal cues and demonstrations, repeatedly reminding participants to maintain a perceived exertion level of 5–8 on the Borg CR10 scale and showing modifications to make each exercise easier or more challenging.

The program consisted primarily of bodyweight exercises, with occasional use of simple household items (e.g., a towel or water bottle) as minimal external resistance. To accommodate different fitness levels, three difficulty levels (low, moderate, high) were available and assigned according to participants’ baseline aerobic capacity from cardiopulmonary exercise testing (CPET). The three difficulty levels were differentiated by increasing physical demand and movement complexity, with higher levels incorporating more challenging and technically demanding exercises. Exercise categories were re-evaluated after 12 weeks based on follow-up CPET results and subjective training feedback. Within each session, participants were encouraged to adjust movement pace or range of motion to match the prescribed perceived exertion and enable gradual progression. All videos were cancer-type-specific excluding movements contraindicated for each entity (e.g., avoiding shoulder loading in breast cancer, prone positions in colorectal cancer, and jumping in prostate cancer).

Participants were instructed to exercise at a perceived exertion level of 5–8 on the Borg CR10 scale, approximately corresponding to 60–90% of HRmax [24]. The Borg CR10 scale and %HRmax show a strong correlation and serve as approximate methods for classifying exercise intensity within a range high enough to improve cardiorespiratory fitness [20, 24, 38]. The target range of 5–8 was chosen to represent moderate-to-vigorous intensity, as recommended by current exercise oncology guidelines [4], ensuring adequate cardiovascular stimulus while minimizing the risk of overexertion in cancer survivors. Providing a range rather than a fixed value accounted for fluctuations in health status common among cancer survivors and reflected the intermittent nature of strength-endurance training, which alternated work and rest periods, unlike continuous steady-state exercise. Participants were educated on the scale before the intervention during the initial participant training on the CRBP-TS system and regularly reminded of the target intensity during sessions. No separate practical familiarization phase was conducted prior to the intervention. During exercise, participants were not blinded to the HR display on their tablets. However, they were asked not to monitor HR during the sessions and were given no information on how to interpret or adjust training based on it. They also received no guidance on the relationship between RPE and HR. A more detailed description of the training protocol and digital infrastructure has been published by our group previously [36].

### Data collection and processing

#### Heart rate

The current gold standard for HR measurements is the 12-lead electrocardiogram (ECG); however, chest straps are accurate for continuous heart rate monitoring during training movements [39]. During each session, HR was recorded using a Bluetooth-connected chest strap (Garmin HRM Dual, Garmin Ltd., Switzerland) that transmitted data continuously to a patient tablet (Lenovo Tab M10 TB-X606X, Lenovo, Hongkong, China) and the server back-end. Before analysis, all HR recordings underwent a standardized plausibility check and preprocessing to maximize data quality and ensure that only physiologically plausible and technically valid HR responses were included in our analyses.

We excluded the first three minutes (warm-up) and the last two minutes (cool-down) of each session to isolate the exercise-specific HR response. Because the chest strap was paired with the tablet rather than the wrist-worn device, several preprocessing steps were required to ensure signal integrity. Data were recorded at 0.5-second intervals and normalized to 1-second intervals to eliminate duplicate values. The resulting time series was divided into consecutive 1-minute blocks. During some sessions, the HR display froze at values between 70 and 79 bpm, frequently at 72 bpm. This was a typical Garmin bug when the device lost the Bluetooth connection to the tablet. To identify these bugs, we calculated the within-block variance. Zero variance was physiologically impossible and was excluded. Before the interventions started, the participants had been adequately trained to avoid other signal issues (hydration, strap contact, motion).

We retained sessions only if (a) they contained more than 15 valid 1-minute blocks, and (b) the total sum of block variances exceeded seven. This threshold was based on prior experience with the dataset and served to exclude flat-line or noisy data caused by signal loss or dropouts. Maximum session duration—including warm-up, rest periods, and cool-down—was limited to 50 min. As all training sessions lasted approximately 30 min by design, this criterion served as an additional quality control to identify and exclude technically corrupted or incomplete recordings.

To express exercise intensity relative to individual capacity, we calculated %HRmax as the mean HR during the session divided by the maximum HR obtained from CPET. Each participant completed a CPET at baseline and again after 12 weeks, using standardized termination criteria [17]. CPET included electrocardiography (BT300 electrocardiogram, custo GmbH, Germany), impedance cardiography (PhysioFlow, Manatec Biomedical, France), and ergospirometry (Dynostics, Sicada GmbH, Germany) to assess HR, VO_2_, and cardiac output conducted on an electronically braked semi-recumbent ergometer. The initial load was 30 W, with increments of 10 W per minute until either subjective or objective exhaustion occurred, or termination criteria were met. The %HRmax value for each session was referenced to the corresponding CPET from the same half of the intervention (weeks 1–12 or 13–24).

#### Rating of perceived exertion

Participants reported RPE immediately after completing each training session, using the tablet interface. To ensure that RPE corresponded to the overall session rather than the most recent effort, participants were instructed to answer the question: “How strenuous did you perceive this exercise session as a whole?”. Evidence suggests that immediate post-exercise ratings are not systematically biased by the final exercise bout and that a 30-minute delay, as previously proposed, is not required [40]. These values were then used for analysis.

#### Intensity discrepancy score

To explore the discrepancy between subjective and objective measures of exercise intensity, we calculated an intensity discrepancy score (ΔIntensity) for each training session. This score was defined as the observed %HRmax minus the predicted %HRmax based on the individual’s reported RPE. Predicted values were derived from the regression model established in Objective 1 (i.e., %HRmax = β₀ + β₁ × RPE). A positive ΔIntensity indicates that the actual HR was higher than expected based on the perceived exertion, suggesting an underestimation of effort. Conversely, a negative ΔIntensity reflects a lower HR than expected, indicating an overestimation of effort. This session-level discrepancy metric allowed for detailed analysis of individual and temporal variation in self-regulation accuracy throughout the intervention.

### Statistical analysis

Sessions with missing RPE or HR data were excluded from the respective analyses. All models were computed based on available cases (listwise deletion per model), allowing us to include participants with missing data. We treated RPE as a continuous variable to increase statistical power. All analyses were performed using IBM SPSS Statistics (version 29.0, IBM Corp., Armonk, NY, USA). Statistical significance was defined as *p* < 0.05 (two-tailed) for all tests. Effect sizes were estimated using marginal and conditional R² values for mixed-effects models.

#### Objective 1: rating of perceived exertion for guidance and monitoring of exercise intensity

To assess the relationship between RPE and %HRmax, we calculated a linear mixed-effects model with %HRmax as the dependent variable and RPE as a fixed effect, with a random intercept modeled at the patient level. Model assumptions, including normality of residuals, homoscedasticity, and linearity, were checked and met. Both %HRmax and RPE were approximately normally distributed, and scatterplots confirmed a linear relationship. We included all sessions with valid %HRmax and RPE data.

Each session was further evaluated based on whether it fell within the subjective target zone of RPE 5–8, corresponding to RPE prescription, and within the target zone of 60–90% HRmax. The lower bound of 60% HRmax reflected the minimal desired training intensity [4, 21], while the upper bound of 90% HRmax was included to account for safety considerations to avoid overexertion in this oncological cohort [4]. Frequencies and proportions were calculated and summarized descriptively. Additionally, we constructed a 2 × 2 contingency table to evaluate how well RPE could identify sessions that were within the physiological target zone. For this analysis, %HRmax was considered the reference standard, and RPE was treated as the test measure. We calculated sensitivity and specificity to quantify how accurately RPE classified sessions as being inside or outside the physiological target zone.

#### Objective 2: predictors of discrepancies between perceived exertion and HR-based intensity

We computed a linear mixed-effects model with ΔIntensity as the dependent variable, including age, sex, training week and beta-blocker use as fixed effects, and a random intercept at the patient level. The variable “training week” represented the sequential week of the 24-week intervention (weeks 1–24) and was included as a continuous variable to model temporal trends in self-regulation accuracy. Due to strong structural overlap between cancer type and sex, cancer type was excluded from the final model to avoid multicollinearity. Model assumptions (normality of residuals, homoscedasticity, and linearity) were examined and met.

## Results

Comprehensive baseline characteristics of the intervention group (*n* = 76) have been described in detail elsewhere [17]. Table 1 summarizes the key characteristics of the intervention group relevant to the present analysis.

**Table 1:** Selected baseline characteristics of the intervention group

Variable	Intervention group (*n* = 76)
Age, years	54.4 ± 11.0
Sex, No. female	45 (59%)
Body mass index, kg/m^2^	26.9 ± 4.5
Colorectal cancer, No. Breast cancer, No. Prostate cancer, No.	10 (13%) 43 (57%) 23 (30%)
HRmax CPET 1, bpm	152 ± 19
HRmax CPET 2, bpm	151 ± 18
VO₂max, ml/kg/min	26.2 ± 6.0
Beta-blocker use, No. Cardiac arrhythmias, No.	7 (9%) 0 (0%)
Baseline cancer-related medication*	22 (29%)

Data are presented as means ± standard deviations or as frequencies with percentages in parentheses

the numbers indicate the first (1) CPET at baseline and the follow-up (2) CPET after 12 weeks

*HRmax* Maximum heart rate, *CPET* Cardiopulmonary exercise test, *VO*₂*max* Maximal oxygen uptake

* estrogen receptor modulator (*n* = 9) or monoclonal antibody (*n* = 2) or aromatase inhibitors (*n* = 8) or chemotherapy (*n* = 3)

A total of 3771 training sessions were recorded over the six-month intervention period. After plausibility checks and data preprocessing, valid RPE data were available for 98.6% (*n* = 3720) of sessions. Valid HR data were available for 94.9% (*n* = 3579) of sessions. Overall, 93.7% (*n* = 3533) of sessions included both valid RPE and HR data. Table 2 presents overall training adherence and intensity data. A detailed analysis of adherence patterns has been published previously by our group [17].

**Table 2:** Training adherence and intensity data

Variable	Value	Sample size (*n*)
Training sessions completed per participant	46 (IQR: 28–68)	76 participants
Training sessions per week	2.1 ± 1.1	76 participants
RPE across sessions	6.1 ± 1.5	3720 sessions
Sessions within target RPE (5–8), %	85	3720 sessions
Mean Training HR, bpm	101 ± 15	3579 sessions
Mean Training HR, %HRmax	66 ± 9	3579 sessions
Sessions within target %HRmax (60–90), %	76	3579 sessions
Peak Training HR, bpm	121 ± 19	3579 sessions
Peak Training HR, %HRmax	80 ± 10	3579 sessions

Data are presented as median (interquartile range, IQR) or means ± standard deviations as appropriate. n refers either to the number of participants or to the number of valid training sessions used for analysis

*RPE* Rating of perceived exertion, *HR* Heart rate, *%HRmax* Mean heart rate during each training session expressed as a percentage of the maximum heart rate obtained from cardiopulmonary exercise testing

### Objective 1: rating of perceived exertion for guidance and monitoring of exercise intensity

**Fig. 1:**
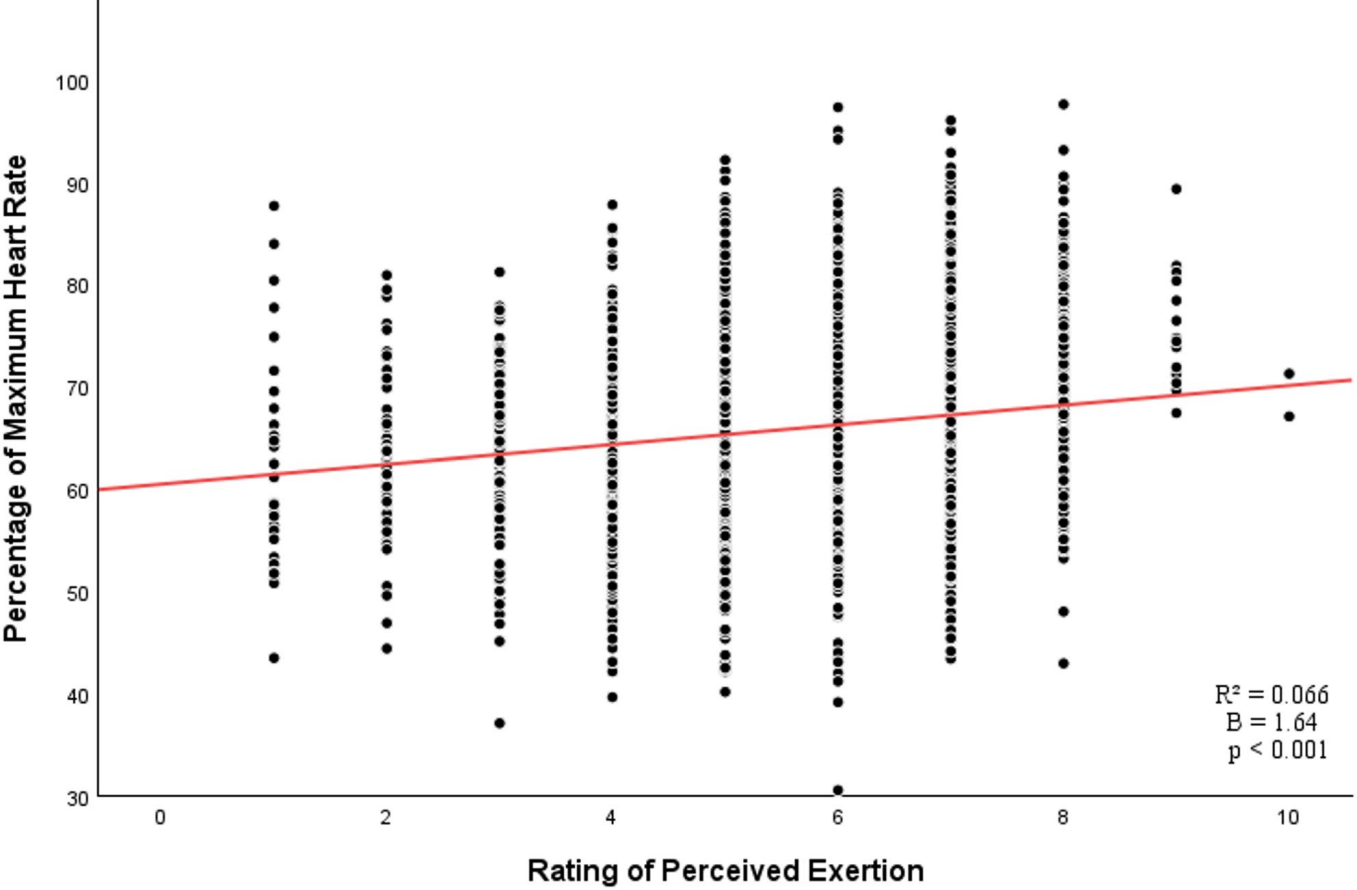
Association between ratings of perceived exertion and percentages of maximum heart rate across 3,533 training sessions. Each point shows a single session. The red line represents the fitted linear regression model for the association

Our linear mixed-effects analysis yielded a statistically significant positive association between RPE and %HRmax (B = 1.64, SE = 0.09, *p* < 0.001; Fig. 1). The marginal R² of the model was 0.066, the conditional R² was 0.622, and the ICC was 0.60.

Across the 3533 sessions with valid RPE and HR data, 66.9% (*n* = 2362) of sessions were within both the target RPE zone (5–8) and the physiological target zone (60–90% HRmax). In 18.2% (*n* = 643) of sessions, RPE was within the target zone, while %HRmax was outside, whereas in 9.7% (*n* = 343) %HRmax was within the target zone, while RPE was outside. Both target zones were missed in 5.2% (*n* = 185) of sessions. Using %HRmax as the reference standard, RPE demonstrated a sensitivity of 87.4% for detecting sessions within the physiological target zone and a specificity of 22.3% for identifying sessions outside the target zone.

### Objective 2: predictors of discrepancies between perceived exertion and HR-based intensity

Our second linear mixed-effects analysis also included all 3533 sessions with complete HR and RPE data. All fixed effects were statistically significant: sex (B = 0.64, SE = 0.06, *p* < 0.001), age (B = 0.037, SE = 0.003, *p* < 0.001), beta-blocker use (B = − 0.39, SE = 0.10, *p* < 0.001), and training week (B = 0.032, SE = 0.003, *p* < 0.001). Both the marginal and conditional R² were 0.101.

## Discussion

In a multicenter home-based rehabilitation trial, we prescribed exercise intensity for each session using RPE while measuring the subjective exertion via RPE and the objective exercise intensity via HR. We analyzed 3533 training sessions completed by 76 participants, representing one of the largest session-based datasets in exercise oncology. To our knowledge, this is the first study to systematically assess the validity of RPE for prescribing and monitoring exercise intensity in cancer survivors engaged in unsupervised, home-based exercise therapy.

By doing so, we aimed to positively direct future research and clinical practice on useful instruments to prescribe and monitor exercise in unsupervised rehabilitation settings.

### Objective 1: rating of perceived exertion for guidance and monitoring of exercise intensity

We found a positive association between RPE and HR during unsupervised, home-based exercise sessions in cancer survivors. On the session level, however, RPE explained only 6.6% of the variance in HR, suggesting that while patients could broadly gauge their exertion, the precision of RPE for reflecting exercise intensity under real-world unsupervised conditions was limited. Notably, the conditional R² of 62.2% indicates that much of the explained variance was attributable to individual-level random effects. Our findings indicate that while the RPE–HR relationship remains fairly consistent within individuals, it differs substantially between them. This makes RPE a useful tool for self-regulation, but less suitable for between-subject comparisons.

Previous studies have reported stronger relationships between RPE and physiological markers of exercise intensity, even in unsupervised home-based settings. For instance, Tang et al. found that RPE explained 30–34% of HR variance during structured endurance training, with stable associations persisting under home-based cardiac rehabilitation [33]. However, their exercise protocol involved standardized aerobic exercise on cycling ergometers and repeated RPE assessments - unlike our asynchronous, multimodal circuit training. Similarly, Aamot et al. found that RPE could effectively guide intensity in cardiac rehabilitation, albeit with inter-individual variability in precision [35]. Scherr et al. further reported strong associations between RPE and HR in a large cohort under laboratory-based treadmill and cycle ergometer testing, with RPE explaining up to 55% of the variance in HR, independent of age, sex, and fitness level [24]. More recently, Ferri Marini et al. highlighted that while RPE-HR relationships established during graded exercise testing can be applied to steady-state exercise, their accuracy decreases with longer durations and higher intensities, emphasizing the limitations of directly transferring laboratory-based RPE-HR relationships to practical exercise contexts [41].

The comparatively lower explained variance in our study likely reflects these contextual and methodological differences. Perceived exertion reflects a combination of central and peripheral signals, but is inherently shaped by individual experience, the type of activity, and the surrounding environment [42]. Our intervention combined unsupervised training, asynchronous video instruction, and session-level RPE reporting - factors that could have contributed to increased variability in perceived exertion.

Compared with supervised or telemonitored programs, the absence of real-time feedback in our asynchronous design likely further reduced RPE precision. In remotely supervised cardiac and oncological rehabilitation, where RPE guidance is complemented by HR feedback or telecoaching, patients tend to maintain target intensity more accurately and show stronger correlations between perceived exertion and physiological load [11, 33, 35]. This suggests that hybrid or feedback-supported approaches may enhance the reliability and safety of self-regulated training in cancer rehabilitation. Our results emphasize the need to further examine the validity of RPE not only in clinical or laboratory contexts but also in complex, real-world oncology rehabilitation settings. In this context, individually calibrating the relationship between RPE and HR may help improve the precision of self-regulation during unsupervised exercise.

Maintaining appropriate exercise intensity is a cornerstone of safe and effective oncological rehabilitation. Especially in early post-surgical phases, insufficient load may limit functional recovery, whereas excessive exertion can increase cardiovascular strain, fatigue, or risk of musculoskeletal injury [4, 43]. Beyond association, we therefore examined whether RPE could identify sessions that met the physiological target zone of 60–90% of HRmax. RPE demonstrated high sensitivity (87%) for detecting target-zone sessions, but low specificity (22%) for identifying sessions outside this range. In total, 66.9% of sessions met both the target RPE and HR zones, and another 9.7% met the HR target despite RPE being outside the recommended range. This suggests that patients using RPE can generally achieve moderate-to-vigorous intensity. This pattern aligns with findings from previous telehealth interventions, where RPE was typically supported by real-time feedback or objective monitoring to enhance accuracy [11]. In fully unsupervised, asynchronous programs like ours, the absence of such feedback may limit RPE’s utility in detecting non-beneficial intensities. Indeed, the low specificity of RPE observed in our study indicates that RPE may not consistently detect when the load is insufficient or excessive, underlining the need for complementary monitoring tools to ensure both safety and training effectiveness of home-based exercise programs. Nevertheless, RPE remains a low-threshold strategy to guide intensity in unsupervised settings, and its utility may improve through individualized calibration or periodic feedback to help patients train at adequate and safe intensities.

### Objective 2: predictors of discrepancies between perceived exertion and HR-based intensity

Because RPE did not consistently align with HR, we examined potential predictors of this discrepancy. In our mixed-effects model, age, sex, beta-blocker use, and training progression all showed statistically significant associations with the difference between RPE and HR. However, all effects were modest, and the overall model explained only 10% of the variance.

Previous studies in healthy and cardiac populations have generally found RPE to correlate well with physiological intensity markers, regardless of age, sex, or medication use [24, 44]. However, these studies were typically conducted under controlled laboratory conditions using treadmill or ergometer protocols. In contrast, our intervention involved cyclical bodyweight-based endurance training with variable movements and pacing, performed in an unsupervised, home-based setting. These modality- and context-specific differences are particularly relevant, given that perceived exertion, though rooted in physiological signals, is known to be influenced by task characteristics, environmental factors, and individual experience [42]. Such influences likely contributed to the greater variability and lower explained variance observed in our study.

We extend existing evidence by showing that in cancer survivors, individual characteristics such as sex or medication use can modestly affect the accuracy of RPE-based self-monitoring. However, these factors explained only a small proportion of the variance. This suggests that perceived exertion during unsupervised training is shaped by additional influences beyond basic clinical or demographic characteristics. Future studies should explore psychological, behavioral, and contextual factors that may help explain the unexplained variability in perceived exertion during unsupervised training.

In interpreting our findings, it is important to consider the respective limitations of both RPE and HR as intensity markers in oncological rehabilitation. While RPE offers a practical and equipment-free means to gauge exertion, it is inherently subjective and does not necessarily parallel physiological strain [45, 46]. Conversely, HR provides an objective physiological indicator, yet its accuracy can be affected by common cancer-related factors such as chemotherapy, endocrine therapy (e.g., fulvestrant, aromatase inhibitors), beta-blocker use, and autonomic dysfunction following surgery or systemic therapy [47, 48]. Given that our participants were 2–6 months post-surgery, such physiological and pharmacological influences likely introduced additional variability and may have contributed to the modest correlation between RPE and HR observed in this study. We therefore emphasize that neither method is flawless, and that combining subjective perception with objective monitoring may offer the most reliable exercise-intensity regulation in cancer rehabilitation.

Taken together, our findings from Objective 1 and 2 suggest that RPE can support the general regulation of exercise intensity in unsupervised, home-based exercise therapy for cancer survivors, helping patients reach recommended intensity ranges. However, RPE alone lacks the precision needed to accurately monitor physiological intensity, as substantial variability remained despite modest contributions from individual factors, such as age, sex, medication use, and training progression. Our findings highlight that while RPE offers a low-threshold approach to prescribing intensity, HR guidance remains important to support precise intensity regulation in unsupervised programs.

Building on this, our results emphasize the need to develop self-regulatory strategies that help cancer survivors better align perceived and physiological intensity during home-based training. In practice, this could include individual RPE calibration during initial supervised sessions, periodic digital feedback on HR–RPE alignment, or hybrid supervision models combining self-guided exercise with remote professional monitoring. Such approaches could improve the accuracy, safety, and sustainability of intensity control while preserving the flexibility that makes digital home-based rehabilitation both accessible and scalable.

### Limitations

This study has several limitations that should be acknowledged. Potential confounders such as treatment-related fatigue, ongoing medication other than beta-blockers, or residual effects of systemic therapy were not systematically recorded and may have influenced both HR and perceived exertion. An exploratory analysis including baseline cancer-related medication is reported in Additional file 1. However, interpretation is limited by strong overlap with sex (21 women, 1 man) and the lack of information on medication changes during the intervention period. In relation to Objective 1, the training protocol involved asynchronous, bodyweight-based circuit sessions with varying exercises, intensities, and environmental conditions. This variability may have affected how participants perceived exertion and reduced the precision of RPE as a standalone tool. Although participants were instructed not to monitor their HR during sessions, some may have done so, potentially influencing their subjective ratings. While our mixed-effects model accounted for interindividual baseline differences, the naturalistic, unsupervised setting did not allow for person-specific modeling of RPE–HR slopes, limiting conclusions about individual calibration needs. Moreover, participants received only theoretical instruction on the RPE scale without a practical familiarization phase, which may have further contributed to interindividual variability in perceived exertion. In addition, prior exercise experience and familiarity with exertion scales were not systematically captured and may influence RPE accuracy, as individuals with greater training experience may show better calibration between perceived exertion and physiological intensity.

With regard to Objective 2, HRmax was determined using a semi-recumbent ergometer, where exhaustion may have been constrained by peripheral fatigue. Since the intervention involved whole-body circuit training with higher muscle mass activation than the CPET modality, calculated %HRmax values may have overestimated actual relative exercise intensity. This could have systematically affected the classification of sessions within or outside the target zone and contributed to discrepancies between RPE and HR. Another limitation is that RPE was assessed only once at the end of each session. This single post-session rating may not fully capture intra-session fluctuations in perceived exertion, particularly in circuit-style training formats with alternating work and rest periods. Collecting RPE at multiple time points during exercise could provide a more nuanced understanding of self-regulation dynamics and improve precision in future studies.

Finally, our sample consisted of cancer survivors selected based on digital competence and relatively high functional capacity after curative surgical and oncological treatment. This may limit the applicability of our findings to individuals with higher symptom burden, lower digital access, or more complex health conditions.

## Conclusions

RPE may serve as a practical, low-threshold tool to broadly guide exercise intensity in unsupervised, home-based training among cancer survivors. However, its limited alignment with HR at the session level in a circuit-style intervention indicates that RPE alone might be insufficient for precise exercise intensity monitoring. Objective measures - such as HR feedback - should complement RPE to ensure adequate exercise intensity in unsupervised settings. Future research should examine individual and contextual factors influencing perceived exertion and test whether the RPE–HR relationship holds across different exercise modalities. In addition, further studies should explore whether individual calibration can improve the accuracy of RPE-based intensity regulation in home-based exercise programs.

## Supplementary Information

Supplementary Material 1.

## Data Availability

The datasets analyzed during the present study can be obtained from the corresponding author on reasonable request.

## Abbreviations

CPET: Cardiopulmonary Exercise Testing
CR10: Category Ratio 10
CRBP-TS: Colorectal, Breast, and Prostate Cancer – Telemonitoring and Self-management Trial
ΔIntensity: Intensity Discrepancy Score
DRKS: German Clinical Trials Register
ECOG: Eastern Cooperative Oncology Group
HR: Heart Rate
HRmax: Maximum Heart Rate
ICC: Intraclass Correlation Coefficient
%HRmax: Percentage of Maximum Heart Rate
RPE: Rating of Perceived Exertion
