# Supplementary material for: Perceived exertion for guiding and monitoring exercise intensity in unsupervised, home-based cancer rehabilitation: an analysis of 3533 exercise sessions
Source: BMC Cancer. 2026 Jan 20;26:130. doi: 10.1186/s12885-026-15588-0 (PMC12836964; doi:10.1186/s12885-026-15588-0)
Supplement: Supplementary file 1 — Supplementary Material 1. [file 12885_2026_15588_MOESM1_ESM.pdf]

Additional File 1

For "Perceived Exertion for Guiding and Monitoring Exercise Intensity in Unsupervised, Home-Based Cancer Rehabilitation: An Analysis of 3,533 Exercise Sessions"

Exploratory analysis including baseline cancer-related medication

To address the reviewer's suggestion, we conducted an exploratory analysis including baseline cancer-related medication (yes/no) as an additional predictor of ΔIntensity. Cancer-related medication strongly overlapped with sex in this cohort (21 women, 1 man), and information on medication changes during the intervention period was not available. Results are therefore reported for transparency and should be interpreted cautiously.

Supplementary table 1 Exploratory analysis of predictors of the discrepancy between physiological intensity and perceived exertion (ΔIntensity), including cancer-related medication

Predictor	B	SE	p-value	95% CI
Age	0.038	0.003	< 0.001	0.033; 0.043
Sex (women vs. men)	0.745	0.066	< 0.001	0.614; 0.875
Beta-blocker use (yes/no)	–0.390	0.097	< 0.001	–0.580; –0.200
Training week (continuous)	0.032	0.003	< 0.001	0.026; 0.038
Baseline cancer-related medication (yes/no)	–0.184	0.064	0.004	–0.309; –0.059
